# Revisiting Glycogen in Cancer: A Conspicuous and Targetable Enabler of Malignant Transformation

**DOI:** 10.3389/fonc.2020.592455

**Published:** 2020-10-30

**Authors:** Tashbib Khan, Mitchell A. Sullivan, Jennifer H. Gunter, Thomas Kryza, Nicholas Lyons, Yaowu He, John D. Hooper

**Affiliations:** ^1^Mater Research Institute—The University of Queensland, Translational Research Institute, Woolloongabba, QLD, Australia; ^2^Faculty of Health, Australian Prostate Cancer Research Centre-Queensland, School of Biomedical Sciences, Institute of Health and Biomedical Innovation, Translational Research Institute, Queensland University of Technology, Woolloongabba, QLD, Australia

**Keywords:** glycogen, cancer metabolism, cancer therapy, metabolic reprogramming, chemoresistance, immunometabolism

## Abstract

Once thought to be exclusively a storage hub for glucose, glycogen is now known to be essential in a range of physiological processes and pathological conditions. Glycogen lies at the nexus of diverse processes that promote malignancy, including proliferation, migration, invasion, and chemoresistance of cancer cells. It is also implicated in processes associated with the tumor microenvironment such as immune cell effector function and crosstalk with cancer-associated fibroblasts to promote metastasis. The enzymes of glycogen metabolism are dysregulated in a wide variety of malignancies, including cancers of the kidney, ovary, lung, bladder, liver, blood, and breast. Understanding and targeting glycogen metabolism in cancer presents a promising but under-explored therapeutic avenue. In this review, we summarize the current literature on the role of glycogen in cancer progression and discuss its potential as a therapeutic target for cancer treatment.

## Introduction

Glycogen is a highly branched polymer of glucose that is used for the efficient storage and release of energy (1). This function is highlighted by the importance of glycogen in organs such as the brain, liver, heart, and muscle (2–5). With critical roles as a feedstock for respiration and biomolecule biosynthesis, it is unsurprising that glycogen also plays an active role in human pathology, driving certain epilepsies in the brain, and contributing to diabetes-induced kidney damage and severe muscle wasting disorders (2, 6, 7). Glycogen is also implicated in malignancy, and glycogen-based metabolic reprogramming has recently received renewed attention as a targetable phenotype in the tumor microenvironment (8, 9).

**Figure 1:**
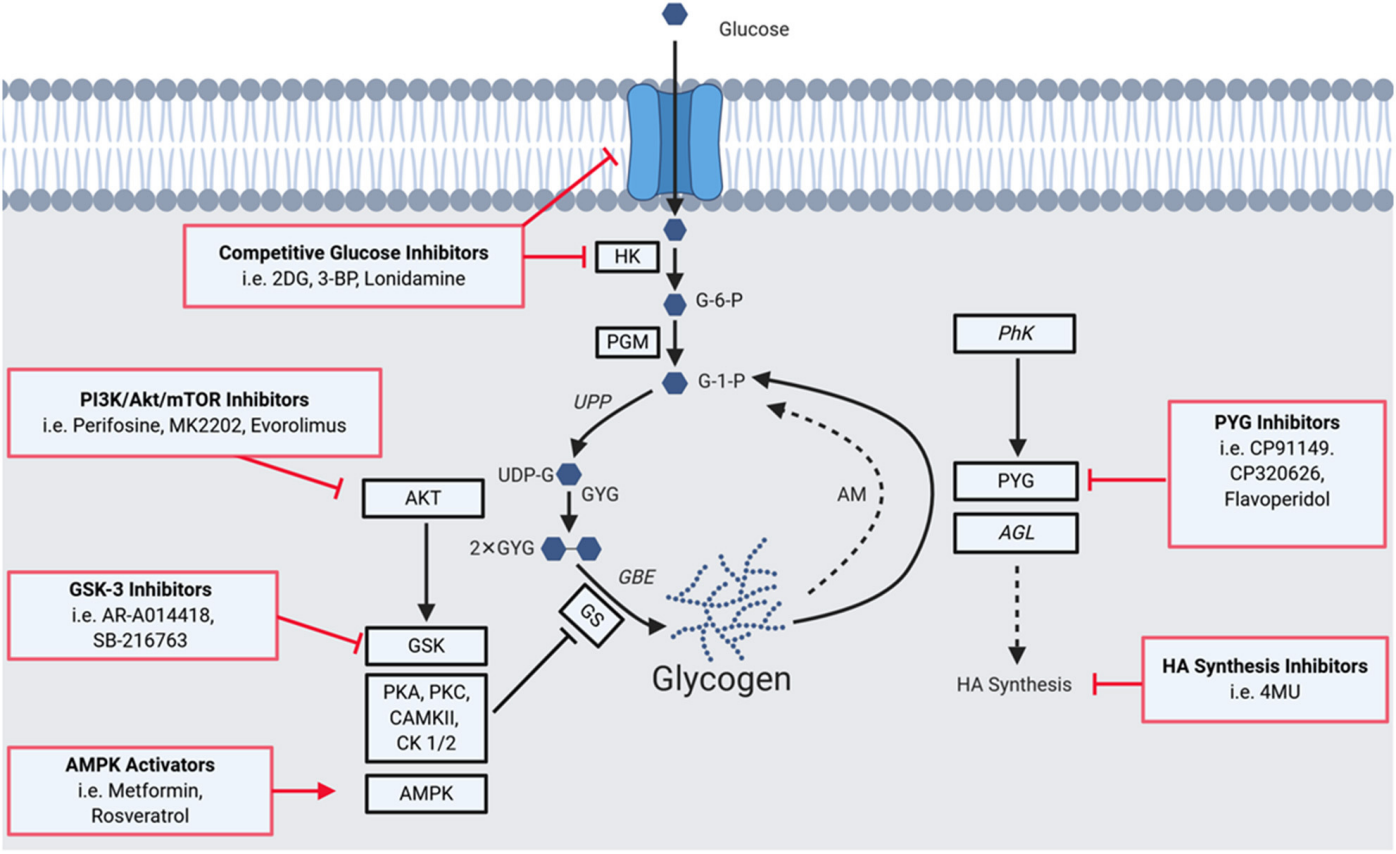
A Summary of the glycogen metabolism axis, including agents that pharmacologically modulate the axis. Glucose enters cells via glucose transporters (GLUTs), where it is phosphorylated to glucose-6-phosphate (G-6-P) by hexokinase (HK), followed by a transfer of the phosphate group from carbon 6 to 1 by phosphoglucomutase (PGM) to yieldglucose-1-phosphate (G-1-P). The resultant G-1-P is converted to UDP-glucose (UDP-G) by UDP-glucose pyrophosphorylase (UPP). This is then used by glycogenin (GYG; in dimerized form), a specialized primer of glycogen synthesis, to auto-glucosylate and extend its chain. After auto-glucosylation, glycogen synthase (GS) elongates these initial chains and creates further α-1,4-glycosidic linkages. Branchpoints of α-1,6-glycosidic linkages are mediated by the glycogen branching enzyme (GBE). GS is heavily regulated allosterically and by a network of kinases. Phosphorylation of GS decreases its activity and hence the rate of glycogen synthesis. GS has phosphorylation sites for Glycogen Synthase Kinase 3 (GSK3), protein kinase A (PKA), protein kinase C (PKC), calmodulin-dependent protein kinase II (CaMKII), AMP-activated protein kinase (AMPK), casein kinase 1 (CK1), and casein kinase 2 (CK2). GS is also positively regulated: allosterically by glucose-6-phosphate, and via dephosphorylation by protein phosphatase 1, regulatory subunit 3 (PPP1R3C). Phosphorylation can also mediate activity with phosphorylase kinase (PhK) activating the enzyme and driving glycogenolysis. A proportion of glycogen is also degraded via the lysosomal autophagy route by the enzyme acid maltase (AM). Enzymes of the pathway implicated in tumorigenesis are highlighted by black squares, those implicated in tumor suppressive roles (PhK and AGL) are italicized. Red boxes indicate means of pharmacologically modulating the axis, highlighting glycogen-targeted drugs that have already been used for clear cell cancers. Glucose entry and processing can be inhibited by 2-deoxy-D-glucose (2DG), 3-bromopyruvate (3BP), and lonidamine. AKT and GSK which inactivate GS by phosphorylation can be targeted with GSK3B inhibitors (e.g., AR-A014418 and SB-2216763), as well as PI3K (Perifosine), AKT (MK2202) and mTOR (Everolimus) inhibitors. AMPK can be activated to promote glycogen synthesis, as has been performed with metformin and resveratrol. PYG has been targeted using inhibitors such as CP91149, CP320626, and flavoperidol, to prevent glycogen mobilization. Furthermore, the downstream effects of AGL on hyaluronic acid synthesis can be disrupted using 4-methylumbelliferone (4MU).

Figure 1 summarizes key aspects of glycogen metabolism and overlays inhibitors that have been employed to disrupt it in disease processes. The first step of glycogen synthesis involves glucose entry into cells via glucose transporters. Upon entry, glucose is phosphorylated to glucose-6-phosphate (G-6-P) by hexokinase (HK), followed by transfer of this phosphate group to carbon 1 by phosphoglucomutase (PGM). The resultant glucose-1-phosphate (G-1-P) is converted to uridine diphosphate glucose (UDP)-glucose by UDP-glucose pyrophosphorylase (UPP). Then, dimeric glycogenin (GYG), a specialized primer of glycogen synthesis, auto-glucosylates UDP-glucose to generate an initial chain of (1 → 4)-α linked glucose units on each of the two glycogenin monomers. After auto-glucosylation, glycogen synthase (GS) elongates these initial chains and creates further (1 → 4)-α glyosidic linkages, recruiting more UDP-glucose as the substrate (9, 10). The creation of branchpoints [(1 → 6)-α glycosidic linkages] is catalyzed by glycogen branching enzyme (GBE).

Glycogen can be degraded to release glucose via two distinct pathways. The first occurs in the cytoplasm and involves the enzymes glycogen phosphorylase (PYG) and glycogen debranching enzyme (AGL). The second is an autophagic lysosomal route via the enzyme acid maltase that recycles ~10% of glycogen, the dysfunction of which gives rise to glycogen storage disease type II, known as Pompe's disease (9, 10).

The enzymes that mediate the rate-limiting steps of glycogen metabolism are GS, for synthesis, and PYG, for degradation (9, 10). GS is negatively regulated by an extensive network of kinases including GS kinase 3 (GSK3), protein kinase A (PKA), protein kinase C (PKC), calmodulin-dependent protein kinase II (CaMKII), AMP-activated protein kinase (AMPK), casein kinase 1 (CK1), and casein kinase 2 (CK2) (2, 9). Conversely, GS is positively regulated by allosteric mechanisms including by the allosteric stimulator G-6-P and via its dephosphorylation by protein phosphatase 1 regulatory subunit 3C (2, 9).

PYGs catalyse the first step of glycogen degradation, glycogenolysis, converting glycogen to G-1-P, which is transformed to G-6-P for energy production. There are three PYG isoforms, that display restricted expression patterns: PYGL (liver), PYGM (muscle) and PYGB (brain). PYG exists as a heterodimer with multiple activity states, and activity can be regulated by both negative and positive allosteric effectors at regulatory sites on each monomer. Phosphorylation can also modulate activity with phosphorylase kinase (PhK) activating the enzyme and driving glycogenolysis (9, 10).

The present work details the mechanisms by which glycogen metabolism is hijacked during cancer progression and summarizes the latest data on visualizing, quantifying, and targeting aberrant glycogen metabolism in cancer.

## Reprogrammed Glycogen Metabolism in Cancer

**Figure 2:**
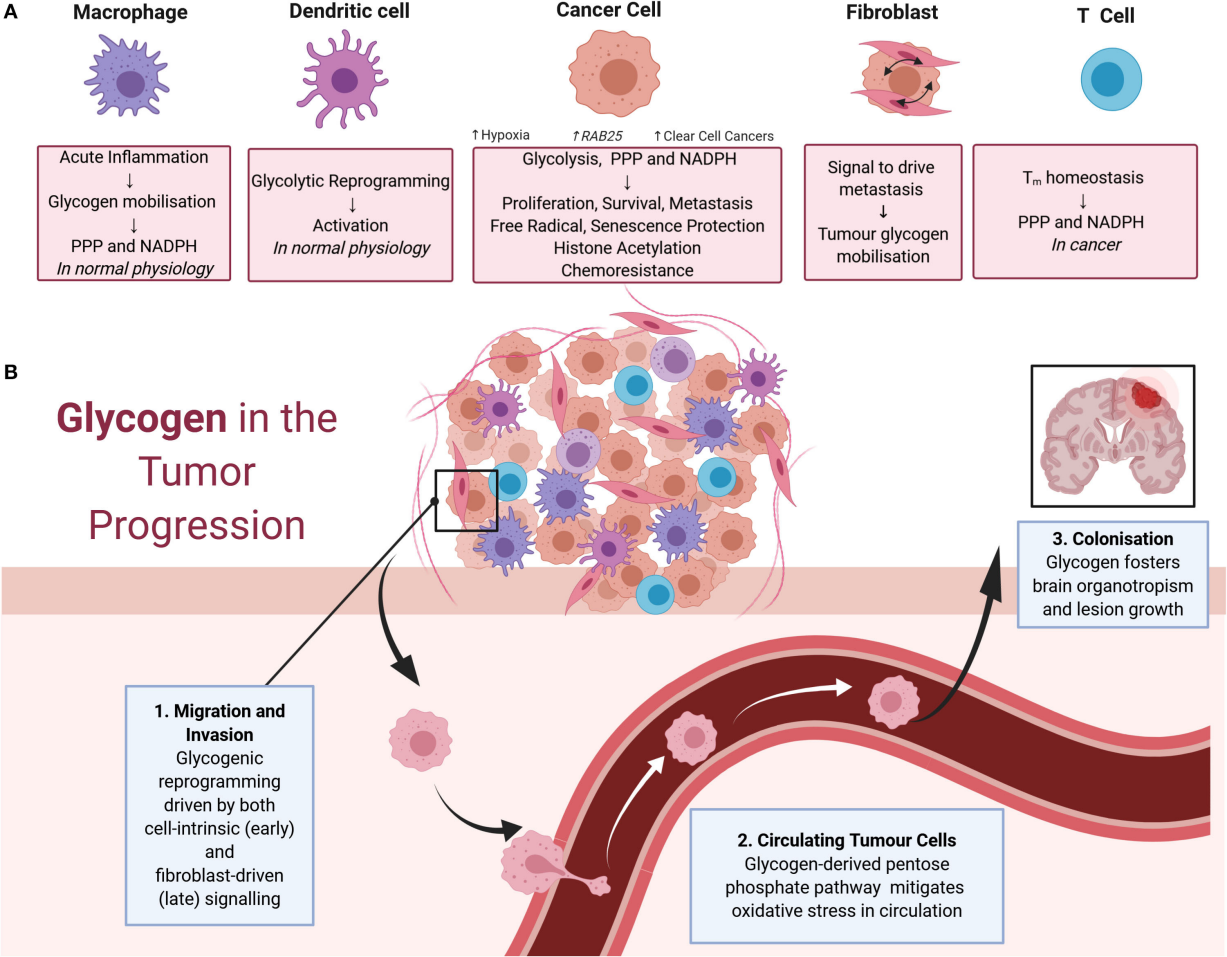
**(A)** Impacts of glycogen on cells of the tumor microenvironment. In macrophages, physiologically acute inflammation causes glycogen mobilization as a feedstock for the pentose phosphate pathway and NADPH production. Glycogen also drives the early stages of dendritic cell activation via glycolytic reprogramming. These physiological processes are also thought to contribute to cancer progression. In cancer cells, glycogen fuels glycolysis, the pentose phosphate pathway, and NADPH production to support cell proliferation, survival, metastasis, free radical protection, cell senescence protection, histone acetylation, and chemoresistance. Tumor glycogen metabolism increases in a variety of scenarios, including in hypoxic conditions and via the induction of the *RAB25* oncogene. Tumor glycogen metabolism is also ubiquitously high in clear cell cancers. In cancer associated fibroblasts, bi-directional signaling between fibroblasts, and neighboring cancer cells drives tumor glycogen mobilization to fuel metastasis. For T cells, glycogen stores consumed via the pentose phosphate pathway for NADPH production are essential for CD8^+^ memory T cell survival. **(B)** Glycogen in tumor progression. Glycogen mobilization is implicated in metastasis including as fuel for: (1) migration and invasion of cancer cells in the early stages of metastasis with crosstalk between fibroblasts and malignant cells also eliciting glycogenolysis to fuel migration and invasion; (2) the pentose phosphate pathway in circulating malignant cells facilitating detoxification of reactive oxygen species and promotion of survival; and (3) colonization of distant sites including in the survival of brain metastases.

The contributions of glycogen metabolism to cancer progression are summarized in Figure 2. The importance of glycogen in carcinogenesis was suggested by a 1981 study demonstrating that it accumulates in a range of carcinomas at levels higher than in surrounding tissues (11). PYG, the key enzyme in glycogenolysis, and upstream and downstream mediators of glycogen degradation, are associated with poor prognosis and diverse malignant phenotypes in a range of cancers (9, 12–17). Interestingly, other enzymes involved in glycogenolysis are thought to have tumor-suppressive roles. This includes the glycogen debranching enzyme AGL which had a suppressing function in models of bladder and lung cancers, and the kinase PhK β-subunit (PHKB) which suppressed models of hepatocellular carcinoma (18–21). Interestingly, these tumor suppressive roles are potentially independent of glycogen metabolism (19, 21). In the case of AGL, this involves the maintenance of amino acid homeostasis and the nucleotide precursor pool, whilst PHKB negatively regulates AKT and STAT3 signaling (18, 19).

The following paragraphs detail examples of the causes and phenotypic consequences of altered glycogen metabolism in cancer.

### Common Mediators of Tumor Glycogenesis

Even before molecular studies on the roles of glycogen in cancer, it was apparent from histological analysis that a sub-set of carcinomas are enriched in glycogen. The malignant cells of these glycogen-enriched tumors display clear cytoplasm that is vacuolated due to the loss of glycogen that results from histological processing (10, 22). These “clear cell” malignancies include cancers of the kidney, ovary, breast, endometrium, and colorectum (23–26). The two most common clear cell cancers—renal and ovarian—have been well-characterized molecularly and histologically and provide useful systems to interrogate the roles of glycogen in tumorigenesis (26–28).

Glycogenesis occurs in response to diverse physiological and pathological stimuli including oxygen and nutrient deprivation (1, 3, 6, 29, 30). In the normal brain, both glial and neuronal glycogen are protective against hypoxia-induced death (29–31). In cancer, the enzymes of both glycogen synthesis and catabolism rise sharply in response to oxygen and nutrient deficiency, suggesting that both glycogenesis and glycogenolysis are important as tumors evolve in response to local and systemic challenges (32, 33). Key mediators of these responses are hypoxia-inducible factors (HIFs) which regulate tumor survival, metastasis, and therapy resistance (34, 35). For clear cell renal cell carcinoma aberrant hypoxic signaling is initiated by loss of the Von Hippel Lindau (VHL) gene, a negative regulator of HIFs. For ovarian clear cell carcinoma, which arises in the hypoxic environment of endometriotic cysts, the molecular aberrations initiating the hypoxic response remain to be elucidated (22, 36–38). Interestingly, oncogene amplification can also be an important mediator in altered tumor glycogenesis. An example is amplification of the *RAB25* gene, which occurs in subtypes of ovarian, renal, prostate, liver, and breast cancer (39, 40). The RAB25 protein is a small GTPase involved in endosomal recycling which interacts with AKT to drive glycogenesis, generating tumor glycogen that can be mobilized in settings of nutrient stress (39). At least in ovarian cancer, RAB25 regulates HIF-1α activity in an oxygen-independent manner, suggesting that RAB25 lies upstream to hypoxia-based induction of glycogen metabolism (41). Despite differences in the molecular initiators and drivers of HIF activity, clear cell cancers employ common mediators of the glycogenesis pathway during malignant transformation, including RAB25, GLUT1, AKT, GSK3, and AMPK, that represent a “glycogen addicted” signature (22, 38, 42, 43).

### Glycogen Driving Carcinogenesis

Several recent molecular studies have contributed to elucidating the functions of glycogen in cancer. A seminal loss of function genetic study targeting PYG in models of glioblastoma, breast, and colon cancer demonstrated that glycogen catabolism is a key driver of cancer cell proliferation, survival, and protection from hypoxia-induced free radicals (12). Depletion of the PYG liver isoform PYGL induced premature cell senescence via a ROS-dependent mechanism, and reduced glucose input into the pentose phosphate pathway (PPP) (10, 12). Cell accumulation of glycogen granules is a hallmark of several physiological processes associated with senescence, including the replicative senescence of primary human fibroblasts and neuronal and hepatic aging (12, 44, 45). In the context of cancer, senescence is often viewed as a double-edged sword, on one hand driving tumor stasis, but on the other providing a means of therapy resistance, and fostering a microenvironment favoring inflammation, invasion, and angiogenesis (44–48). Further understanding the role of glycogen in cancer senescence may provide a window into modulating this fine balance (49, 50).

Interestingly, PYG via a mechanism involving the E3 ubiquitin ligase malin, can also translocate to the nucleus to drive nuclear glycogenolysis non-small cell in lung cancer, fueling compartmentalized pyruvate production and histone acetylation (51). Aberrant histone acetylation resulting in an altered epigenetic landscape is a hallmark of multiple cancers including non-small cell lung cancer (52, 53). Depletion of malin impairs nuclear glycogenolysis by blocking nuclear translocation of PYG. The resulting accumulation of nuclear glycogen is reversed by re-introduction of malin in lung cancer cells which restores nuclear catabolism of glycogen, increases histone acetylation, and decreases growth of non-small-cell lung cancer xenografts in mice (51). This highlights the importance of glycogen beyond a cytoplasmic glucose reservoir, revealing this macromolecule as a key driver of compartmentalized tumor metabolism.

### Glycogen and the Immune Compartment: Potential Pro-tumoral and Anti-tumoral Roles?

Aberrant immune system responses, including those mediated by metabolic reprogramming, are integral to cancer progression (54, 55). As summarized in Figure 2A, dendritic cells (DCs), macrophages, and T cells have been implicated in metabolic reprogramming in cancer. Mobilization of cell-intrinsic glycogen fuels glycolytic reprogramming and subsequent immune activation of DCs (56–58). For macrophages, glycogen metabolism regulates M1-mediated acute inflammatory responses by fueling the pentose phosphate pathway to generate NADPH for survival and also potentially facilitating the breakdown of immunological tolerance in cancer settings (59). Similarly, maintenance of CD8^+^ memory T (Tm) cells requires glycogenolysis to fuel the pentose phosphate pathway, generate NADPH, and promote oxidative homeostasis (60). Consistent with this role of glycogen metabolism in immune system responses, disruption of glycogenolysis in the Tm cell compartment caused growth of subcutaneous melanoma cell xenografts and reduced survival of tumor-bearing mice (60). Disruption of the glycogen axis is thought to impair nascent anti-tumor immunity to drive cancer progression and also inhibit immunotherapeutic responses mediated by T-cells (61, 62). Here, it is important to note that immune system responses can elicit both pro- and anti-tumoral actions, and metabolic reprogramming underlies this fine balance (54, 63, 64). Thus, it is possible that global disruption of glycogen metabolism as a therapeutic approach will have adverse consequences for cancer patients because of unwanted effects on the immune compartment. However, it appears that this is not an unassailable problem because metabolic targeting has progressed in the past despite effects on both malignant cells and the immune system. For example, inhibitors of the monocarboxylate transporter MCT1 have progressed to clinical trials despite early concern about deleterious effects on the T cell division that is essential for effective immune response (65, 66). In fact, MCT1 inhibitors have since been shown to improve the infiltration of anti-tumor immune cells (67). Similar progress with glycogen-directed therapies will require a greater understanding of the distinguishing metabolic features of immune and malignant cells, with the key perhaps lying in developing rational combinations of therapeutic agents that selectively synergize in cancer cells (54).

### Glycogen as a Metastatic Fuel

Death from cancer is due largely to metastatic rather than primary tumor burden (68, 69). As summarized in Figure 2B, the multistep processes required for metastasis are facilitated to varying degrees by cancer-induced reprogramming of glycogen metabolism. Vascular and lymphatic metastasis involve release of malignant cells from the primary tumor, intravasation, dissemination via the vasculature or lymph, extravasation at secondary sites, establishment of a blood supply, and evasion of internal and external cues to undergo cell death, then thriving in the foreign environment (68–70). The early stages of this metastatic cascade, involving degradation of extracellular matrix (ECM) and local invasion involves HIF-mediated metabolic reprograming that drives acidification of the extracellular space and glycolysis including from glycogen stores (69). In models of breast cancer and osteosarcoma mobilization of glycogen stores by brain glycogen phosphorylase PYGB promotes migration and invasion (71, 72). Also, in pro-metastatic hepatocellular carcinoma, metabolic reprogramming toward glycogenesis, in particular involving UPP, is a key mediator of metastasis (73).

Metastasis also requires the interaction of cancer cells with the cells of the tumor microenvironment including fibroblasts (69). Cancer-associated fibroblasts (CAFs) can mobilize cancer cell glycogen to drive metastasis in models of ovarian cancer (74). It is mediated by the p38α MAPK pathway in CAFs releasing soluble factors to act on malignant cells. This reprogramming event is particularly important in stages of metastasis involving growth of cells in suspension. Cells exposed to death-inducing levels of oxidative stress are protected by ROS-detoxifying metabolites generated via glycogen catabolism feeding the pentose phosphate and one-carbon metabolism pathways, which release NADH, NADPH, and glutathione (74, 75). Glycogen is an important fuel to overcome oxidative stress, allowing cancer cells to successfully survive in suspension during this step of metastasis.

The final stage of metastasis, successful colonization of other organs (68, 69), presents a considerable challenge to malignant cells as the microenvironment at the distant site can be hostile. As a result, metastasizing cancer cells display a propensity to disseminate to particular organs, a relationship called organotropism, that display favorable environments including metabolic requirements (68, 69). There are indications of such an organotrophic relationship between glycogen rich cancers and brain metastases. Notably, glycogenesis sustains glucose-independent growth and survival of breast cancer cells metastasized to the brain (76). In this regard, it was interesting to note that glycogen-rich clear cell carcinoma of the breast displays a higher proportion of brain metastases than other subtypes of breast cancer (23). These findings are perhaps unsurprising, given the roles of glycogen in brain physiology, including neuronal and glial growth, and pathology, including certain epilepsies (31, 77, 78). However, based on the difficulty associated with effective treatment of brain metastasis, targeting of glycogen could perhaps provide a new treatment avenue. There are also suggestions that glycogen mobilization facilitates omental metastases of certain peritoneal tumors, such as ovarian, endometrial serous, colon, gastric, and pancreatic lesions (74), thus inhibition of glycogen catabolism could benefit these patients (79).

### Glycogen and Chemoresistance

The importance of glycogen to chemoresistance is highlighted by the observation that glycogen-rich clear cell carcinomas are distinguished by higher chemoresistance than non-clear cell malignancies (43, 80, 81). Clear cell ovarian carcinomas are generally more resistant to conventional platinum and taxol-based chemotherapies compared to other ovarian cancer histotypes, and clear cell renal cell carcinomas rapidly acquire resistance to standard of care tyrosine kinase inhibitors as well as other targeted therapies (43, 81–83). Consistent with this, increased activity of key mediators of glycogen metabolism (glucose, AKT, GSK, and AMPK) is strongly associated with chemoresistance (84–86). Mechanistically, it appears that glycogen-derived metabolites contribute to chemotherapy resistance via a variety of mechanisms, including via ATP-based drug efflux and ROS protection, however more work is required to understand these processes (87).

## Visualizing and Quantifying Tumor Glycogen

The size and abundance of glycogen facilitate its visualization and use to delineate glycogen-dependent tumors. Hematoxylin and eosin, and periodic acid-Schiff stains are used in routine histopathology assessments to delineate clear cell tumors (9) and antibodies are available to immunohistochemically distinguish glycogen from lipids and glycoproteins (51, 88). Glycogen granules can also be readily detected by electron microscopy, and this technique has been used in studies to detect carcinogenesis-associated changes in glycogen content (10, 12, 32, 60, 74).

The ability to label glycogen with glucose and glucose analogs has facilitated labeling strategies to measure glycogenic flux. Compounds such as 2-[N-(7-Nitrobenz-2-oxa-1,3-diazol-4-yl)amino]-2-deoxy-D-glucose (2NBDG) that readily incorporate into glycogen have provided a relatively cost-effective way to determine broad cellular glycogen content (89). Similarly, glycogen content can be assessed in cell and tissue homogenates spectrophotometrically by taking advantage of degradation of glycogen to glucose which is detected and quantified using luminescent or fluorescent substrates (90, 91). Glycogen content can also be quantified by incorporation of ^13^C-labeled glucose (6, 8, 25, 51, 71). Similarly, radiolabeling with ^18^F-N-(methyl-(2-fluoroethyl)-1H-[1,2,3]triazole-4-yl)glucosamine (^18^F-NFTG) is being used to specifically detect oncogene-driven changes in glycogenesis in *in vivo* preclinical models (92). ^18^F-NFTG overcomes certain limitations of FDG PET imaging including high cardiac uptake and low differentiation between neoplastic and inflammatory tissue (92). Clinical use of this tracer could be informative in defining distant metastases, although high background signal associated with glycogen-rich organs such as the kidney and liver presents a challenge that requires mitigation. ^18^F-NFTG may also provide opportunities for a theranostic-like approach to patient care involving delineation of glycogen-containing tumor burden followed by treatment with glycogen-targeted pharmacological agents. The marriage of metabolic imaging and targeting is beginning to bear fruit for glutamine-addicted (^18^F-FGln), fat-addicted (^11^C-acetate), OxPhos-addicted (^18^F-BnTP), and glycolytic (^18^FDG) tumors, although it is important to note that radiotracer uptake may not consistently correlate with the activity of the targeted metabolic process in all settings due to the interconnectedness of metabolic pathways (68, 93–95).

## Pharmacological Disruption of Glycogen Metabolism in Cancer

The importance of glycogen in a range of processes required for cancer progression suggests that disrupting glycogen metabolism is a means to therapeutically target cancer, in particular tumors displaying glycogen dependence such as clear cell malignancies. Figure 1 highlights several points at which the glycogen axis has potential to be pharmacologically targeted, including the entry of glucose into cells, and during various catalytic processes required for glycogenesis and glycogenolysis. An example of a pharmacological disruptor of glycogen metabolism is the glucose analog 2-deoxy-D-glucose (2DG) which competes with glucose for cell entry via GLUT transporters and as a phosphorylation substrate for hexokinase (96–98). 2DG incorporation into glycogen antagonizes glycogenolysis, and glycogen mediates 2DG resistance via the activity of brain glycogen phosphorylase isoform PYGB (98–101). It has recently been shown that 2DG synergizes with carboplatin against patient-derived mouse models of glycogen-rich ovarian clear cell carcinoma (95). This strategy to increase the efficacy of a key standard-of-care chemotherapy was achieved at a 2DG dose far lower than employed in other studies against non-glycogen dependent cancers (97). This raises the possibility that combining 2DG and chemotherapy could be effective for clear cell cancers generally, including the much more common clear cell renal cell carcinoma (96, 102). The data justify further clinical trials to evaluate the efficacy of these combinations in these glycogen-dependent cancers.

Disruption of glycogen metabolism is also achieved by targeting mediators of glycogen biosynthesis including the PI3K/AKT/mTOR pathway. Modulators of this pathway, such as perifosine, MK-2202, and evorolimus, have progressed into clinical trials, however, it is not yet clear whether anti-cancer effects are mediated by impacts on tumor glycogen metabolism (103, 104). Hyperactivation of the pathway is a prominent molecular feature of both clear cell ovarian and renal cell carcinomas (37, 42, 43, 105). PI3K, AKT, and mTOR inhibitors have shown promise in these malignancies as standalone therapies and in combination with standard-of-care chemotherapies, displaying synergistic anti-proliferative effects *in vitro* and *in vivo* (17, 106–109). Furthermore, inhibitors of GSK3β have been pursued as standalone treatments and in combination with multi-kinase inhibition in clear cell renal cell carcinoma, where it potentiates the effects of sorafenib both *in vitro* and *in vivo* (110–113).

In certain cancer contexts, it also appears to be useful to promote rapid glycogenolysis to exhaust glycogen stores. For example, myeloid leukemia cells display pathological glycogenesis via a mechanism involving suppression of AMPK activity, with the accumulated glycogen available for mobilization as a glycolytic fuel during times of stress (17). These processes can be targeted with AMPK activators such as metformin and resveratrol, anti-diabetic agents that are becoming more widely studied in cancer (9, 114, 115). Metformin functions by inducing glycogenolysis via insulin signaling, downstream of GSK activity, as well as by AMPK activation (9, 17, 116–118). Pharmacological disruption of processes downstream the glycogen debranching enzyme AGL has also been attempted. This involved via modulation of the hyaluronic acid synthesis axis using drugs such as 4-methylumbelliferone (4MU) which reduced progression of bladder and lung cancers in model systems (19, 21, 119).

Inhibition of glycogenolysis has been most commonly attempted in cancer settings via disruption of PYG (9, 120). PYG inhibitors are often repurposed anti-diabetic therapies which target different allosteric sites of PYG (9) and produce similar phenotypes to genetic depletion of PYG, including impaired glycogenolysis resulting in cell cycle arrest, apoptosis, and ROS-dependent cell senescence (12, 121, 122). CP-320626, an inhibitor of AMP allosteric regulatory and catalytic sites of PYG induces cell cycle arrest and apoptosis in cultured MIA pancreatic cancer cells via MAPK/ERK and TNF-α/NF-κB pathways (123). Interestingly, flavoperidol, a potent inhibitor of CDK activity that progressed to clinical trials, has recently been shown to produce similar effects to genetic disruption of PYG and to be a bona-fide PYG inhibitor (21, 124, 125). The most widely used indole carboxamide PYG inhibitor CP-91149 induces growth inhibition in a range of tumors including hepatocellular carcinoma and pancreatic and prostate cancer (121, 126, 127). It acts synergistically with standard-of-care multi-kinase inhibitors sorafenib and regorafenib in models of hepatocellular carcinoma (121). It will be important to assess the efficacy of a similar regime in models of clear cell renal cancer, for which multi-kinase inhibitors are also standard-of-care. Despite promising *in vitro* results, PYG inhibitors are yet to be evaluated in *in vivo* tumor models.

Interestingly, disruption of enzymes involved in glycogen metabolism induces cell death by apoptotic and non-apoptotic processes including necroptosis and ferroptosis (128). PYGL inhibition induces necroptosis by modulating ROS production and aerobic respiration (129, 130). Similarly, an upstream regulator of PYG, phosphorylase kinase G2, is critical to the lipid peroxidation underlying ferroptosis (128, 131). To optimize the efficacy of glycogen metabolism disruptors against cancer, including reducing therapy resistance, further research is required to better understand the mechanisms by which these agents induce cell death. Additional research is also required to minimize adverse events that can occur when targeting metabolic processes which are important in both normal and malignant cells. This is highlighted by toxicities that have been reported from trials of 2DG against a range of advanced solid tumors, where the clinically tolerable dose of this agent was accompanied by significant adverse events including cardiac and gastrointestinal toxicities and neutropenia (28, 29), with one patient experiencing a fatal cardiac arrest (96, 102).

## Conclusion

Despite the key role of glycogen in processes that underpin malignant transformation, there are still many gaps in our understanding of the molecular mechanisms that regulate metabolism of this macromolecule in cancer. The importance of glycogen metabolism to tumor cell senescence, metastasis, both in driving the metastatic cascade and organotropism, and in resistance to chemotherapies and targeted therapies, are aspects that warrant particular attention. New information in these areas is expected to lead to further attempts to harness glycogen as an effective therapeutic target (8). It seems likely that the repurposing of glycogen metabolism targeting agents that are currently used for non-cancer pathologies including diabetes, will benefit patients with glycogen-dependent clear cell cancers and potentially other non-clear cell malignancies.

## Author Contributions

TKh wrote the manuscript. MS, JG, TKr, NL, YH, and JH edited the manuscript. All authors contributed to the article and approved the submitted version.

## Conflict of Interest

The authors declare that the research was conducted in the absence of any commercial or financial relationships that could be construed as a potential conflict of interest.
